# Supplementary material for: Safety and Immunogenicity of a Carbohydrate Fatty Acid Monosulphate Ester Adjuvant Combined with a Low-Dose Quadrivalent Split-Virion Inactivated Influenza Vaccine: A Randomised, Observer-Blind, Active-Controlled, First-in-Human, Phase 1 Study
Source: Vaccines (Basel). 2024 Sep 10;12(9):1036. doi: 10.3390/vaccines12091036 (PMC11435821; doi:10.3390/vaccines12091036)
Supplement: Supplementary file 1 [file vaccines-12-01036-s001.zip › vaccines-3140233-supplementary.pdf]

Supplementary information

Safety and Immunogenicity of a Carbohydrate Fatty Acid Monosulphate Ester Adjuvant Combined with a Low-Dose Quadrivalent Split-Virion Inactivated Influenza Vaccine: A Randomised, Observer-Blind, Active-Controlled, First-in-Human, Phase 1 Study

Valentino D'Onofrio [1], Sharon Porrez [1], Bart Jacobs [1], Azhar Alhatemi [1], Fien De Boever [1], Gwenn Waerlop [1], Els Michels [2], Francesca Vanni [3], Alessandro Manenti [3], Geert Leroux-Roels [1], Peter Paul Platenburg [4], Luuk Hilgers [4] and Isabel Leroux-Roels [1]

Affiliations

[1] Center for Vaccinology, Ghent University and Ghent University Hospital, 9000 Ghent, Belgium

[2] Harmony Clinical Research BV, 9090 Melle, Belgium

[3] VisMederi S.r.l., 53035 Siena, Italy

[4] LiteVax, 4061 BJ Ophemert, The Netherlands

Corresponding author:

Isabel Leroux-Roels

isabel.lerouxroels@uzgent.be

Supplementary methods

Humoral immune response

Five mL of blood were collected by venous puncture in serum separation blood collection tubes (Becton Dickinson Vacutainer tubes) from all participants at baseline (Day 0) and at Day 7, Day 28 and Day 180. Serum was collected after centrifugation for 10 minutes at 1300-2000g and frozen at 400µL per aliquot.

Humoral immune response was evaluated through Haemagglutination Inhibition (HI) Assay and live virus Micro-Neutralization assay using an ELISA-based read out (MNE). In the HI Assay, all serum samples were pre-treated with receptor destroying enzyme (RDE) (ratio 1:5) from Vibrio Cholerae (Sigma-Aldrich) for 18 h at 37° C in a water bath, then heat inactivated for 1 h at 56° C in a water bath with 8% sodium citrate (ratio 1:4). Serum samples were pre-diluted 1:10 with 0.9% saline solution, then in a 96-well plate, 2-fold serially diluted in ten consequent wells. Each serum sample was tested in duplicate. Twenty-five microliters of standardized antigen (A/Victoria (H1N1) 20/232, A/Darwin (H3N2) 21/318, B/Phuket 21/136 and B/Austria 21/316 all provided by NIBSC), were added to each well and the mixture was incubated at room temperature for one hour. Turkey Red Blood Cells (RBCs) were centrifuged two times, washed with 0.9% saline solution, and adjusted to a final dilution of 0.35%. Fifty microliters of RBCs were added in each well and, after one hour of incubation at room temperature, the plates were visually evaluated for the presence of agglutination inhibition. The antibody titre was expressed as the reciprocal of the highest serum dilution showing complete inhibition of agglutination.

For the MNE assay, heat inactivated serum samples were pre-diluted 1:10, then 2-fold serially diluted in ten consequent wells in a 96-well plate. A standardized amount (100 tissue culture infective dose 50% (TCID50)) of live A/Victoria (H1N1), A/Darwin (H3N2), B/Phuket or B/Austria influenza virus (provided by NIBSC) was added to each well and mixed, then plates were incubated for 1 hour at 37 °C and 5% CO2 in a humidified atmosphere. After the incubation period, 1.5x103 MDCK cells (ATCC) were added in each well to the virus-serum mixture. The plates were then incubated for 16-20 hours at 37°C and 5% CO2 in humidified atmosphere. After overnight incubation, wells were washed, and cells were fixed using cold fixative acetone for 10-12 minutes. Primary antibody against virus N protein was diluted and added to each well: Anti-Influenza A Antibody, nucleoprotein, clone A1 (Millipore) was used for A/Victoria (H1N1) and A/Darwin (H3N2); Mouse Anti Influenza B Monoclonal Antibody (Bio-Rad) was used for B/Phuket and B/Austria. Wells were washed and goat anti-mouse IgG HRP-conjugated

secondary antibody (Sigma-Aldrich) was used. Substrate solution was prepared and added to plates and incubated 10 minutes RT to develop a colorimetric reaction. Stop solution was added and plate optical densities (ODs) were immediately evaluated using an automatic ELISA reader equipped with 490nm wavelength. Each plate had four Cell Control (CC) wells showing no infection, and four Viral Control (VC) wells. Average ODs from CC and VC were used to calculate the infection cut-off value, namely the OD value at which 50% MDCK cells were infected. Based on this, the reciprocal of the highest serum dilution corresponding to the 50% of protection against virus infection represents the neutralization antibody titre for the tested sample.

Both in HI and MNE assays, a positive control having high antibody response for the homologous strain (Sheep antisera A/Victoria (H1N1) 21/120, A/Darwin (H3N2) 21/324, B/Phuket 19/322 and B/Austria 21/326, all provided by NIBSC) and a negative control were used as assays internal quality controls.

Cell-mediated immune response

PBMC isolation and ICS analyses were done according to the protocol previously described (1). Fifty mL of blood were collected by venous puncture in heparin coated blood collection tubes (Becton Dickinson Vacutainer tubes of 10 mL coated with lithium heparin) from all participants at baseline (Day 0) and at Day 7, Day 28 and Day 180. After 1:2 dilution in Hanks buffered salt solution (HBSS), PBMCs were isolated by density gradient centrifugation (Lymphoprep™), washed twice in HBSS, suspended in freezing solution (10% dimethyl sulfoxide/90% fetal bovine serum v/v), frozen at a concentration of 6 up to 20 million cells/mL and stored in liquid nitrogen until use in the subsequent dedicated assays. PBMCs were thawed and suspended in RPMI 1640 medium supplemented with Minimum Essential Medium non-essential amino acids, L-glutamine, penicillin/streptomycin, sodium pyruvate, 2-mercapto-ethanol (all from Invitrogen), and fetal bovine serum (Seradigm). Then, PBMCs were incubated *in vitro* with the relevant vaccine antigens (HA/H3N2/Darwin/2021, HA/H1N1/Victoria/2019, HA/H0N0/Austria/2021, HA/H0N0/Phuket/2013, and NP/Ann Arbor (H2N2) (PepMix™, JPT Peptide Technologies GmbH, Germany) or left unstimulated (background condition with 0.32% DMSO) in the presence of costimulatory antibodies to CD28 and CD49d. Brefeldin A, a protein transport inhibitor, was added after 2 hours for subsequent overnight culture. The following day, cells were stained using fluorochrome-conjugated antibodies to phenotypic markers (CD3, CD4, and CD8), activation (CD40L)

and cytokine (interferon g [IFN-g], interleukin 2 [IL-2], and tumor necrosis factor a [TNF-a]) markers. The samples were analysed by flow cytometry (BD LSR Fortessa X-20, FlowJo v9.9.6).

Influenza-specific CD4+ and CD8+ T cells were determined by flow cytometry as the CD3+CD4+ and CD3+CD8+ events expressing one marker or a combination of markers of CD40L, IFN-g, IL-2, and TNF-a after *in vitro* stimulation with the vaccine antigen, from which the corresponding signal of the same sample obtained in the background condition was subtracted. The intracellular cytokine staining (ICS) results were reported as the frequencies (%) of influenza specific CD4+ or CD8+ T cells per parent population. Results below 0.0001% after background subtraction were set at 0.0001%. All analyses have been done with ICS data multiplied by 10,000, resulting in the frequency of cells per million parent cells.

1. Begue S, Waerlop G, Salaun B, Janssens M, Bellamy D, Cox RJ, et al. Harmonization and qualification of intracellular cytokine staining to measure influenza-specific CD4(+) T cell immunity within the FLUCOP consortium. Front Immunol. 2022;13:982887.

Supplementary Table S1. In- and exclusion criteria.

Inclusion criteria	
1	Written signed informed consent obtained before any study-related activities.
2	Aged 18 to 50 years inclusive, at the time of signing the ICF.
3	Participants who are considered to be in good general health as determined by medical evaluation including medical history, physical examination and laboratory tests within 21 days prior to enrollment.
4	Participants with a BMI within the range 18.5 to 35 kg/m2 inclusive at screening.
5	Women who are not pregnant or breastfeeding, and one of the following conditions applies:<br>• Women of non-childbearing potential (WONCBP).<br>Non-childbearing potential is defined as surgically sterilized (e.g. hysterectomy, bilateral oophorectomy, or tubal ligation/salpingectomy) or postmenopausal (defined as having no menstrual bleeding for at least 12 months) without an alternative medical cause prior to study.<br>OR<br>• WOCBP and using a highly effective contraceptive method (with a failure rate of less than 1 % per year) from at least 1 month prior to study vaccination and for 3 months post-vaccination.<br>The investigator should evaluate the potential for contraceptive method failure (e.g., noncompliance, recently initiated) in relationship to study vaccination. Highly effective contraception is defined as stabilized on oral birth control for at least 1 month before study participation, intrauterine device/system, implant, injection, transdermal patch, vasectomized partner, or sexual abstinence (excluding periodic abstinence). The reliability of sexual abstinence needs to be evaluated in relation to the duration of the study and the preferred and usual lifestyle of the participant. The participant should commit her abstinence to at least 1 month prior to study vaccination and for 3 months post-vaccination. If the participant will not maintain abstinence and changes her status, the participant must first commit to another highly effective method of contraception, which should be discussed with the investigator prior to terminating sexual abstinence as contraceptive method.
6	WOCBP must have a negative serum pregnancy test at screening and a negative urine pregnancy test before vaccination at Day 1. The investigator is responsible for review of medical history and menstrual history to decrease the risk for inclusion of a woman with an early undetected pregnancy.
7	Participants who are willing and able to comply with the study procedures and are in the view of the investigator capable of completing the study.
Exclusion criteria	
1	History of previous laboratory confirmed influenza infection in the past 12 months, excluding laboratory confirmed COVID-19 infections, prior to the day of study vaccination.
2	Positive (in the past, suspected or ongoing) for hepatitis B surface antigen (HBsAg), hepatitis C virus (HCV) antibody, and human immunodeficiency virus (HIV) antibody.
3	Past or current history of immune mediated and/or autoimmune diseases as indicated by the investigator, e.g., diabetes mellitus (type I or II, with the exception of gestational diabetes) and thyroid disease.

4	Serious reactions to vaccines that preclude receipt of study vaccinations as determined by the investigator.
5	Clinical conditions representing a contraindication for IM administration, as judged by the investigator, e.g., history of bleeding disorder (e.g., factor deficiency, coagulopathy, or platelet disorder requiring special precautions) or significant bruising or bleeding difficulties with IM administration or blood draws.
6	History of confirmed hypersensitivity, allergy and/or anaphylaxis to eggs (ovalbumin or chicken proteins), squalene-based adjuvants, or other components of the study vaccine (neomycin, formaldehyde, or octoxinol-9).
7	Current history of uncontrolled medical illness (unstable for the past 3 months) as indicated by investigator, e.g., hypertension.
8	Past or current history of any neurological disorder, e.g., Guillain-Barré syndrome and seizure disorder other than: 1) febrile seizures, 2) seizures secondary to alcohol withdrawal more than 3 years ago, or 3) seizures that have not required treatment within the last 3 years.
9	History of asplenia, functional asplenia or any condition resulting in the absence or removal of the spleen.
10	Active malignancy or malignancy within the past 5 years.
11	Asthma that is unstable or required emergent care, urgent care, hospitalization or intubation during the past two years or that is expected to require the use of oral or intravenous corticosteroids.
12	History of hereditary angioedema (HAE), acquired angioedema (AAE) or idiopathic forms of angioedema.
13	History of idiopathic urticaria within the past year.
14	History of heavy smoking, drug - or alcohol abuse/addiction (including alcohol dependence), or psychiatric condition (e.g., past or present psychoses; disorder requiring lithium; or within 5 years prior to administration of study vaccine, a history of suicide plan or attempt), which in investigator's opinion could compromise the participant's safety and/or compliance with the protocol.
15	A rash, dermatological condition or tattoos that would, in the opinion of the investigator, interfere with injection local reaction rating.
16	Prior seasonal or pandemic influenza vaccination in the 6 months before administration of study vaccine or planning to receive the influenza vaccination during the study period.
17	Prior receipt of investigational pandemic influenza vaccine in the 3 months before administration of study vaccine or planning to receive such product during the study period.
18	Prior receipt of a live attenuated vaccine in the 28 days prior to administration of study vaccine, or within 14 days for subunit or inactivated vaccines other than seasonal or pandemic influenza vaccination, excluding COVID-19 vaccine.
19	Prior receipt of COVID-19 vaccine in the 7 days before administration of study vaccine or planning to receive a COVID-19 vaccine during the first 14 days following study vaccination.
20	Planning to receive a vaccine during the first 28 days following the administration of study vaccine, other than COVID-19 vaccine.

21	Currently participating in another clinical study or planning to participate in another study during the study period, or administration of any investigational drug or medical device in the 4 weeks prior to study vaccination.
22	Prior receipt of blood, blood-derived products, or immunoglobulins in the 6 months prior to administration of study vaccine or planning to receipt such product during the study period.
23	Use of drugs that can affect immune response such as systemic corticosteroids (excluding topical preparations and inhaled preparations) or immunosuppressive drugs in the 30 days before study vaccination and/or in the first 28 days following study vaccination, with the exceptions that a short course of corticosteroids ≤10 days duration, or a single injection for a self-limited condition at least 2 weeks prior to enrollment will not exclude study participation.
24	Current intake of drugs that increase bleeding risk, e.g., anticoagulant medication (coumarin derivatives, low molecular weight heparin, DOAC).
25	Current anti-tuberculosis prophylaxis or therapy.
26	Use of non-steroidal anti-inflammatory drugs (NSAIDs) and/or planning a medical procedure under full anesthesia on the day of study vaccination and/or in the first 14 days following study vaccination with the exception of a medical indication.
27	WOCBP who are pregnant, breast-feeding or planning to become pregnant during the study.
28	Participants with history of any medical conditions that, in opinion of the investigator, might interfere with the results of the study or pose additional risk to the participants due to participation in the study.
29	Current febrile illness (oral temperature >38.0 °C) or other acute illness prior to vaccine administration. Participants with oral temperature >38.0 °C can be rescheduled to when they are at least 72 hours feverless as long as the delayed visit and randomization are still within the screening window defined by protocol.
30	Intake of antipyretics and/or analgesic medications within 24 hours prior to study vaccination. Reason for use (prophylaxis or treatment) should be documented. Participants can be rescheduled after being 24 hours free of intake of antipyretics and/or analgesic medications as long as the delayed visit and randomization are still within the screening window defined by protocol.

Supplementary Table S2. Protocol-defined Safety Lboratory tests.

<table>
<tr><th>Laboratory Tests</th><th colspan="2">Parameters</th></tr>
<tr><td rowspan="7">Haematology</td><td colspan="2">• Platelet count</td></tr>
<tr><td colspan="2">• RBC count</td></tr>
<tr><td>• RBC indices:</td><td>– Mean corpuscular volume (MCV)
– Mean corpuscular haemoglobin (MCH)</td></tr>
<tr><td>• RBC</td><td>– % Reticulocytes</td></tr>
<tr><td>• White blood cell (WBC) count with differential:</td><td>– Neutrophils
– Lymphocytes
– Monocytes
– Eosinophils
– Basophils</td></tr>
<tr><td colspan="2">• Haemoglobin</td></tr>
<tr><td colspan="2">• Haematocrit</td></tr>
<tr><td>Biochemistry</td><td>• Urea
• Creatinine
• Glucose (nonfasted) (only screening)
• HgbA1c (only screening)</td><td>• Aspartate aminotransferase (AST)
• Alanine aminotransferase (ALT)
• Alkaline phosphatase
• Total and direct bilirubin
• C-reactive protein (CRP)</td></tr>
<tr><td rowspan="3">Viremia (only on screening)</td><td colspan="2">• HBsAg</td></tr>
<tr><td colspan="2">• HCV antibody</td></tr>
<tr><td colspan="2">• HIV antibody</td></tr>
<tr><td rowspan="3">Urinalysis (only on screening)</td><td colspan="2">• Glucose</td></tr>
<tr><td colspan="2">• Protein</td></tr>
<tr><td colspan="2">• RBC</td></tr>
<tr><td>Pregnancy testing</td><td colspan="2">• Highly sensitive serum (only screening) and urine β-human chorionic gonadotropin (β-hCG) (Vaccination visit day 0) pregnancy test (as needed for WOCBP)</td></tr>
</table>

Supplementary Table S3. Geometric mean microneutralization antibody titres (GMTs), per cohort per vaccine strain at baseline and 7, 28, and 180 days after vaccination.

		**VaxigripTetra (15 µg)**				**VaxigripTetra (3 µg) + 0.5 mg CMS**				**VaxigripTetra (3 µg) + 2 mg CMS**			
		A/Darwin (H3N2)	A/Victoria (H1N1)	B/Austria	B/Phuket	A/Darwin (H3N2)	A/Victoria (H1N1)	B/Austria	B/Phuket	A/Darwin (H3N2)	A/Victoria (H1N1)	B/Austria	B/Phuket
**Day 0**	GMT (95% CI)	8.6 (6.6 - 11.2)	72.1 (27.4 – 190.0)	8.4 (5.8 - 12.2)	100.2 (58.9 - 170.6)	9.7 (7.0 – 13.3)	107.4 (34.9 – 330.9)	6.3 (4.7 - 8.3)	141.7 (73.4 - 273.6)	9.8 (7.3 - 13.2)	255.5 (111.1 – 587.3)	6.0 (5.1 - 7.2)	119.2 (72.7 - 195.4)
**Day 7**	GMT (95% CI)	13.0 (8.7 – 19.4)	859.2 (384.6 – 1919.5)	21.8 (9.8 - 48.7)	214.8 (111.0 - 415.6)	17.4 (12.6 – 24.0)	801.7 (309.9 – 2074.2)	16.5 (9.4 - 29.2)	283.4 (151.6 - 529.8)	15.4 (11.4 – 20.9)	1631.4 (818.0 – 3253.8)	12.3 (7.1 - 21.4)	444.8 (277.4 - 713.1)
	GMR (95% CI)	1.5 (1.1 – 2.1)	11.9 (4.2 – 33.7)	2.6 (1.2 - 5.0)	2.1 (1.3 - 3.6)	1.8 (1.4 – 2.3)	7.5 (2.9 – 19.4)	2.6 (1.6 - 4.4)	2.0 (1.3 - 3.0)	1.6 (1.2 – 2.0)	6.4 (3.4 – 12.1)	2.0 (1.3 - 3.2)	3.7 (2.3 - 6.0)
**Day 28**	GMT (95% CI)	18.3 (12.2 – 27.6)	1445.1 (699.2 – 2986.4)	45.2 (18.0 - 113.1)	361.3 (215.0 - 607.1)	25.1 (17.1 – 36.8)	1280.0 (619.7 – 2634.9)	40.0 (21.8 - 73.4)	460.5 (239.9 - 883.7)	23.0 (15.6 – 33.7)	2079.4 (974.5 – 4436.8)	40.0 (20.7 - 77.2)	452.5 (263.9 - 776.0)
	GMR (95% CI)	2.1 (1.5 – 3.1)	20 (6.2 – 64.3)	5.4 (2.4 - 12.1)	3.6 (2.0 - 6.5)	2.6 (1.8 – 3.8)	11.9 (3.6 – 39.7)	6.4 (3.5 - 11.5)	3.2 (1.8 - 5.8)	2.3 (1.7 – 3.3)	8.1 (4.1 – 16.3)	6.6 (3.7 - 11.9)	3.8 (2.0 - 7.2)
**Day 180**	GMT (95% CI)	13.4 (9.2 – 19.6	563.3 (296.2 – 1071.1)	31.6 (15.5 - 64.1)	218.2 (125.4 - 379.6)	16.8 (12.0 – 23.6)	1039.7 (480.3 – 2250.8)	40.7 (25.8 - 64.3)	293.4 (164.9 - 522.1)	15.3 (11.6 – 20.7)	1256.9 (550.9 – 2867.7)	51.6 (27.4 - 97.4)	350.6 (218.7 - 562.0)
	GMR (95% CI)	1.5 (1.1 – 2.0)	7.0 (2.9 – 17.0)	3.7 (2.2 - 6.2)	2.0 (1.2 - 3.3)	1.7 (1.3 – 2.3)	9.7 (3.2 – 29.4)	6.5 (3.9 - 10.7)	2.1 (1.4 - 3.1)	1.6 (1.3 – 2.0)	5.2 (2.3 – 11.4)	8.6 (4.9 - 15.2)	2.8 (1.7 - 4.6)

GMT: geometric mean titre; GMR: geometric mean ratio compared to Day 0; 95%CI: 95% confidence interval.

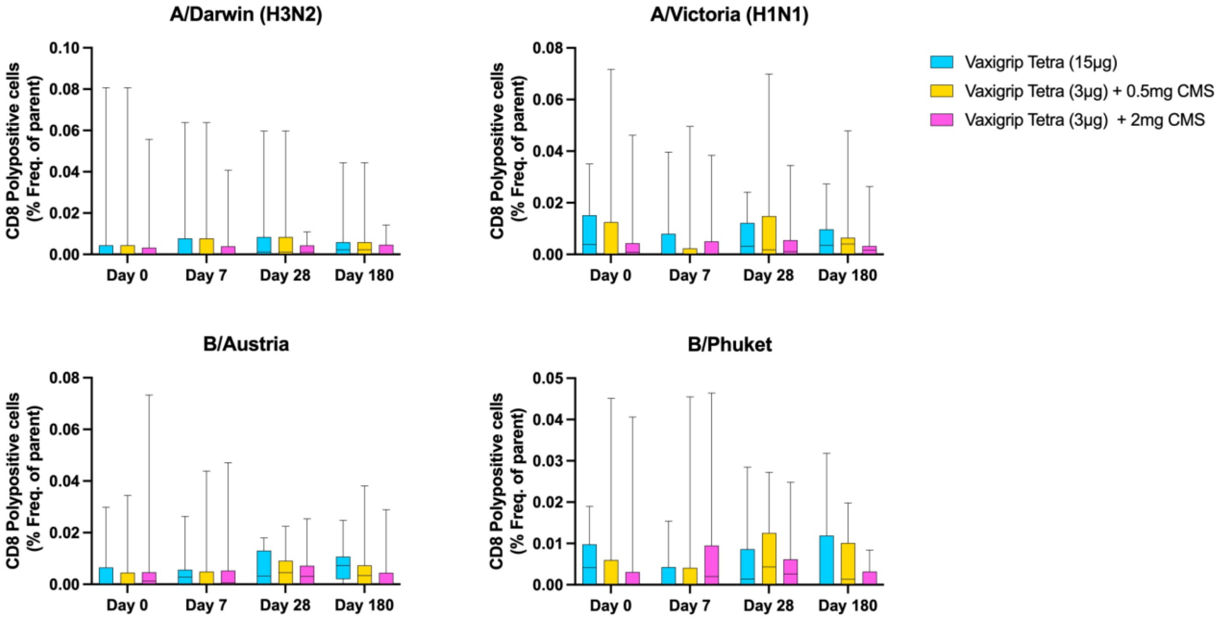

Supplementary Figure S1. Frequency of CD8+ polypositive T cells per cohort per vaccine strain at baseline and 7, 28, and 180 days after vaccination.
